# Caregiver's Opinions on the Design of the Screens of a Future Gamified Mobile Application for Self-Management of Type 1 Diabetes in Children in Saudi Arabia

**DOI:** 10.1155/2021/8822676

**Published:** 2021-02-04

**Authors:** Demah M. Alsalman, Zahra Bu Ali, Zainab Alnosaier, Norah Alotaibi, Turki M. Alanzi

**Affiliations:** Health Information Management and Technology Department, College of Public Health, Imam Abdulrahman Bin Faisal University, Saudi Arabia

## Abstract

The objective of this study was to design the screens of a future gamified mobile application for self-management of type 1 diabetes in children based on the opinion of caregivers at the King Fahad Hospital Diabetes Center, Saudi Arabia. To achieve this objective, a questionnaire was designed and distributed among 100 caregivers through face-to-face communication and social media using a Google Forms link. 65% of the participants met the inclusion criteria. The main result of this study was the design of 13 screens of a gamified application for self-management of type 1 diabetes in children from Saudi Arabia. The key features of the screens were caring for a character; using a challenging friend; inclusion of points, level, and leaderboard as rewarding principles; use of reminders and notifications for doctor's appointments, insulin injection times, blood glucose readings; and tips for improving medication adherence, increasing blood glucose readings, supporting physical activities, and adopting healthy eating habits. It can be concluded that the practical implementation of the screens in a future mobile application can motivate children with type 1 diabetes to improve eating habits, physical exercise, and cognitive, emotional, and social behaviors to maintain a stable state of health. Also, the content of the designed screens can help to monitor blood glucose readings and comply with medication treatment. The designed screens are adapted to the Arab culture.

## 1. Introduction

Diabetes is a chronic disease that prevents the human body from using the glucose contained in food normally [1]. In this state, the pancreas cannot secrete the hormone insulin that helps the body's cells take glucose from the blood [1]. There are 3 types of diabetes: type 1 diabetes, type 2 diabetes, and gestational diabetes (GDM) [2]. Type 1 diabetes occurs more often in children and adolescents; under these conditions, the body makes little or no insulin [2]. Type 2 diabetes tends to occur in adults; when people have this type of diabetes, the body does not use the insulin it produces properly [2]. Gestational diabetes (GDM) can appear in women during pregnancy and can lead to complications in the mother and child; in general, GDM diabetes tends to disappear after pregnancy [2]. If diabetes is not controlled, it can lead to serious complications such as loss of vision and kidney function, stroke, heart disease, foot ulcer, and other illnesses [1].

According to the International Diabetes Federation (IDF), around 463 million people (20-79 years) were living with diabetes worldwide in 2019 [3]. This number will increase to 700 million in 2045 [3]. Furthermore, IDF statistics suggested that in 2019, the countries of the MENA (Middle East/North Africa) region with the highest number of children and adolescents (0-19 years) with type 1 diabetes were Algeria (33,100), Morocco (30,200), and Saudi Arabia (27,800) [3]. Another study pointed out that Saudi Arabia had one of the highest rates of diabetes among countries in the world in 2016 [4]. According to this report, the approximate population of people with diabetes or prediabetes was more than 10 million in 2016 [4]. In 2014, out of a total healthcare budget of 180 billion (Saudi Riyal), 25 billion was spent on patients with diabetes, approximately [4]. This impressive growth rate of diabetes is due to several factors such as obesity, sedentary lifestyle, lack of physical exercise, late diagnosis, lack of consciousness, poor eating habits, high cost of treatment, environmental factors, and genetic influences [4, 5].

There are several alternatives to try to tackle this increasing number of diabetic patients. One of the most important options is to improve diabetes self-management education [6, 7]. Diabetes self-management education is a method that helps patients acquire more knowledge about the impact of the disease on their health [6, 7]. Therefore, the patient can make decisions about treatment options to control the disease tailored to personal requirements [6, 7]. This approach has been used in the self-management of chronic diseases such as type 1 diabetes [6].

It should be noted that one of the methods used to stimulate self-education is through the gamification approach [6, 7]. In recent times, this approach has received significant attention because it has been beneficial in stimulating self-control and active participation of patients in the management of various chronic diseases [6, 7]. However, it is pertinent to indicate that there are not enough published studies that fully confirm the efficacy of gamification [8–10].

The gamification methodology applies the features and processes involved in game mechanics in nongame situations [8]. The purpose of gamification is to involve and engage people to insert enjoyment into common activities to get cognitive, emotional, social, and motivational changes [8]. The use of gamification in the healthcare sector is relatively new, and it influences patient behavior based on the engagement and entertainment of game strategies [9].

Currently, the self-education approach combined with gamification techniques is being used in the development of mobile applications for the management of diabetes [6, 11–13]. Regarding the applications for the management of type 1 diabetes, there are numerous applications available in the commercial market [14–20]. These applications have been designed by companies, institutions, and different kinds of professionals [14–20].

On the other hand, several authors have suggested that many of these applications do not meet the criteria required for medical applications and have been designed without any scientific support derived from medical practice [6, 21, 22]. Besides, some of them do not present evidence of clinical effectiveness or indicate the potential impacts on the health, safety, and confidentiality of patients [6, 21, 22].

Regarding Saudi Arabia, despite the high rate of penetration and use of smartphones, the Internet, and social networks, it was observed that a gamified mobile application for self-management of type 1 diabetes has not yet been developed in this country [23]. In this context, the main objective of this study was to design the screens of a future gamified mobile application for self-management of type1 diabetes in children based on the opinions of patients' caregivers at the King Fahad University Hospital Diabetes Center, Saudi Arabia.

It is worth mentioning that caregivers are the people who care for people with diabetes and may include parents, siblings, relatives, and other guardians [24]. Caregivers help people with diabetes keep medication schedules, doctor appointments, blood sugar levels, exercise activities, dietary control, insulin administration, record glucose readings, check toenails, observe oral problems, and other health complications [24].

In this study, which is the first stage of the application design, we will find out the opinion of the caregivers about the design of the mobile application screens. In future studies, the design and implementation of the mobile application that will help caregivers and physicians control and mitigate the huge burden of diabetes in Saudi Arabia will be completed. The design and implementation of the future mobile application will be based on the scientific criteria of the research team of the Faculty of Public Health, Imam Abdulrahman Bin Faisal University, Saudi Arabia.

## 2. Methods

### 2.1. Study Settings and Participants

To achieve the objective of this study, a questionnaire was designed and distributed among the caregivers of children with type 1 diabetes at the King Fahad Hospital Diabetes Center, Saudi Arabia. The participants were invited through social media using a Google Forms link and through face-to-face communication. The study target was 100 caregivers of children with type 1 diabetes. The response rate was 68%, and 65% of the respondents met the inclusion criterion. The study was approved by the University Institutional Review Board (IRB) Committee at Imam Abdulrahman Bin Faisal University in Dammam, Saudi Arabia. The IRB number was IRB-UGS-2018-03-277.

### 2.2. Inclusion and Exclusion Criteria

The participants selected in this study were caregivers who had children 7 years of age or older at the King Fahad Hospital Diabetes Center, Saudi Arabia. The rest of the participants were excluded.

### 2.3. Description of the Questionnaire

The questionnaire used to obtain the opinion of the caregivers on the design of the screens of the gamified application for children with type 1 diabetes is described in Appendix 1. To design the questions, the commercial gamified applications for self-management of type 1 diabetes in children published in a recent study were analyzed [25]. Next, the characteristics most used in these applications were selected and adapted to our study. Furthermore, based on the criteria of the research group, other important characteristics that were not found in the reviewed applications were added. With this global information, we designed the final questions included in the questionnaire.

The questionnaire consisted of 13 questions. The first part contained 4 questions directed to obtain the demographic information of the participants: (1) gender, (2) age, (3) level of education, and (4) kinship relationship with the child. The second section had 3 questions about the demographic information of the children with diabetes: (1) gender, (2) age, and (3) when the child was diagnosed with diabetes? The third part consisted of 6 questions focused to get information on the design of the mobile application: (1) in your opinion, how much your child with diabetes would benefit from an application that is designed to manage his/her condition? (not helpful, helpful); (2) in your opinion, what is the most appropriate design of the game for your child with diabetes? (questions and answers, runner game, taking care of a character, other); (3) in your opinion, what is the most appropriate rewarding style that should be used in the game? (points, levels, leaderboards, other); (4) what are the behaviors that you struggle to change in your child to manage his/her condition? (physical activities, managing the medication, nutrition problems, monitoring the blood glucose level, other); (5) please rate on a scale from 0-10, how much would your diabetic child benefit from an application designed to manage his\her diabetes condition: not helpful (0)–Very helpful (10)?; and (6) suggest any other comment.

### 2.4. Validation and Reliability of the Questionnaire

The designed questionnaire was validated by 3 experts who were teachers-physicians with a general knowledge of diabetes apps. Also, after developing the questionnaire, a pilot test with 10 participants was carried out. The results of the pilot test were consistent ensuring the reliability of the questionnaire.

### 2.5. Data Collection

The process of developing and distributing the questionnaire among the caregivers of children with type 1 diabetes took three weeks during March 2019. The questionnaire was distributed through face-to-face communication, WhatsApp, and Twitter. It is pertinent to mention that all the concepts and features of the app were explained to the participants face to face and through social media.

### 2.6. Statistical Analysis

A basic descriptive statistical analysis was used to estimate the frequency of the data (percentages). The data was automatically processed and analyzed through Google Forms.

## 3. Results

The demographic information of the caregivers of children with type 1 diabetes appears in Table 1. The majority of respondents were female (98.5%), and most of them were mothers (72.3%). Furthermore, more than half of the respondents (60%) had a college degree level of education, and (72.4%) of the participants were under 40 years of age.

Likewise, Table 2 illustrates the demographic information of children with type 1 diabetes. More than half of them (53.8%) were female, and the majority (70.8%) were under 12 years of age. Most of the participants (84.6%) were diagnosed with diabetes when they were less than 6 years old.

Concerning the opinions of the caregivers on the design of the screens of the mobile application, Table 3 suggests that 29.2% of the respondents thought that the most appropriate game design for the application was taking care of a character. Similarly, the majority of the caregivers (61.5%) believed that the level was the best rewarding style design for the application. Also, more than half of the caregivers (61.5%) reflected that the most suitable feature for the application was challenging friends. Besides, 61.5% of the caregivers found difficulties with changing the nutrition behaviors of their diabetic child, and 55.4% of the caregivers struggled with monitoring the blood glucose level of the child.

Regarding the level of utility of the application measured on a scale from 0 (not useful) to 10 (very useful), Table 4 shows that 39% of the caregivers selected the tenth scale to assess to what extent the application would benefit children in controlling their diabetes condition.

On the other hand, the details of the designed mobile application's screens, in the Arabic language, are shown in Figures 1-13.

**Figure 1:**
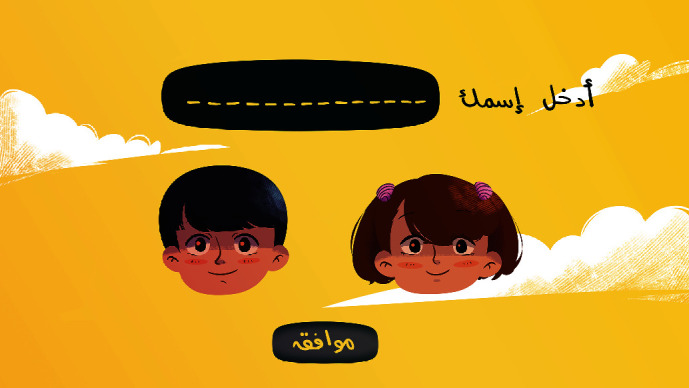
App login screen.

Figure 1 shows the first screen of the application. It is the app login screen. As a player, the child must enter his\her name and chooses the character that reflects his\her gender. Then, the child must click the OK button to proceed to the next screen.

**Figure 2:**
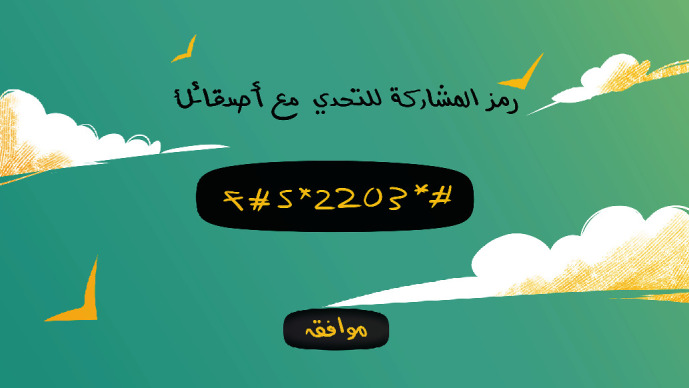
The unique code screen.

Figure 2 displays the second screen that appears after clicking the OK button. This screen displays a code that will be assigned to each player so that he/she can use it to add friends to their list. This code will remain fixed on the home screen as each player will need to enter the same unique code each time they want to log into the game.

**Figure 3:**
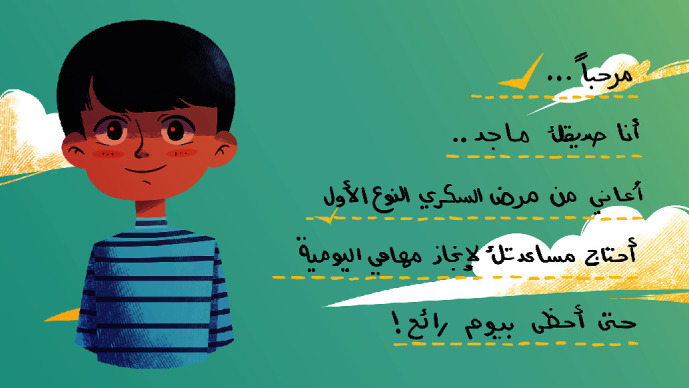
Welcome message screen.

Figure 3 shows a welcome message that the character (he/she) gives the child for joining the game. The message says “Hello, I am your friend ... I suffer from Type 1 Diabetes Mellitus and I need your help in my daily routine activities to have an amazing day.”

**Figure 4:**
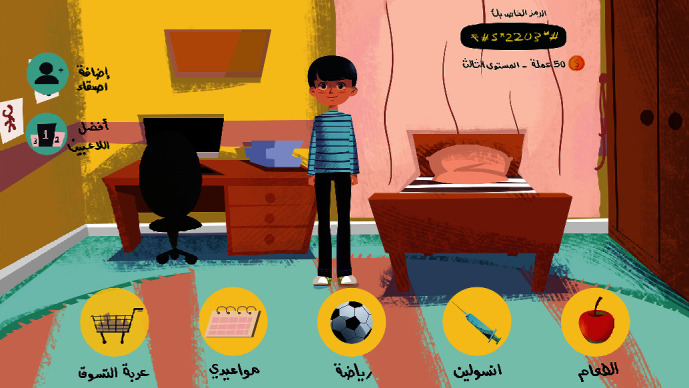
Home screen.

**Figure 5:**
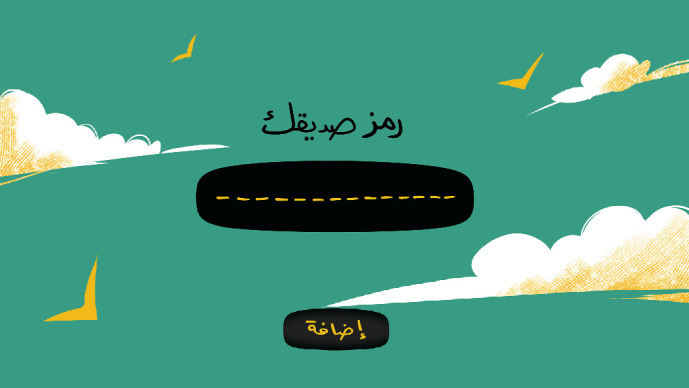
Adding friends screen.

**Figure 6:**
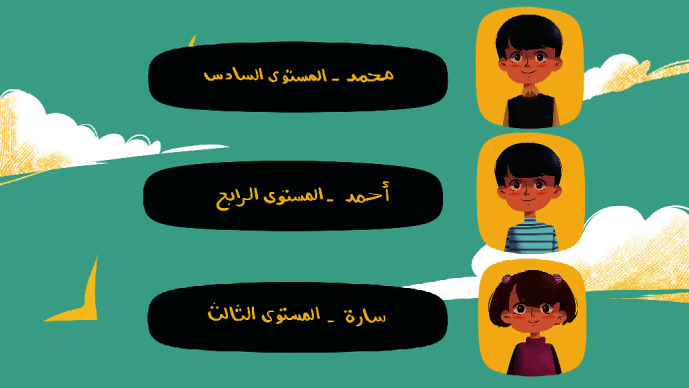
Friends' ranking screen.

Figure 4 illustrates the home screen. This screen shows the character in the bedroom with some icons that take the player to different screens to play different activities. Besides, the upper right part of the screen shows the unique code assigned to the player, the player's level, and the points he accumulates. At the top left side of the screen, there are two icons. One icon shown in Figure 5 is a screen designed to add friends on the player's list using the code. The other icon displayed in Figure 6 illustrates a screen that presents the ranking of the friends on the player's list. Moreover, the player can change the decorations on the home screen by buying accessories from the shopping cart with the points collected.

**Figure 7:**
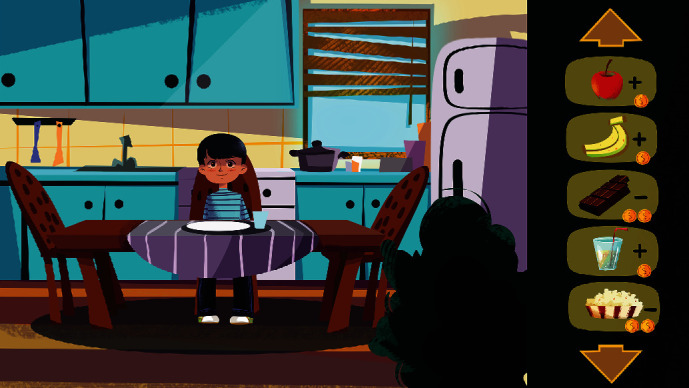
Food screen.

Figure 7 is the food screen that will appear when the player clicks the food icon on the home screen. This screen includes different types of food that the player can use to feed the character. Each type of food can increase or decrease the number of points of the player depending on how healthy the food is for the child with type 1 diabetes. For example, when the child feeds the character an apple, the child gains points and when the child feeds the character chocolate, the child loses points.

**Figure 8:**
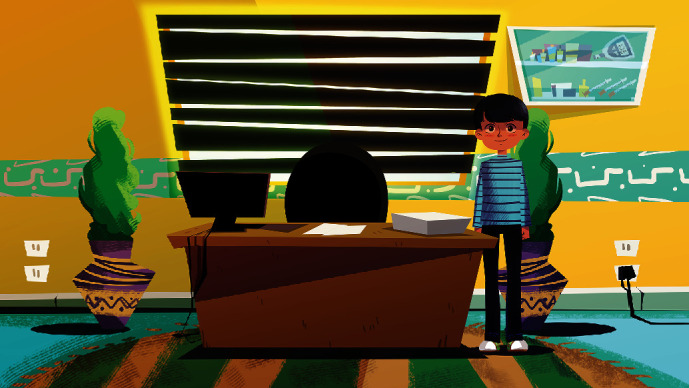
Insulin screen.

Figure 8 is the insulin injection screen that will appear when the player clicks the insulin icon on the home screen. On this screen, the player can take the character's blood glucose reading, which may vary depending on the food the child gives the character. Additionally, this screen includes insulin injections that the player can use to administer insulin to the character.

**Figure 9:**
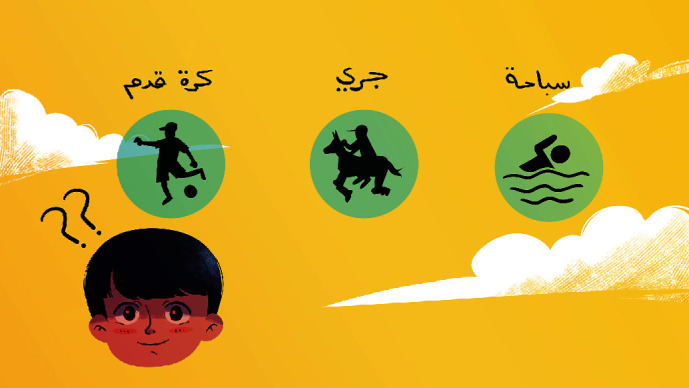
Physical activities screen.

Figure 9 is the physical activities screen that will appear when the player clicks the sports icon on the home screen. This screen highlights the importance of physical activity in the daily routine of diabetic children. There are three icons on the screen for different types of physical activities such as running, swimming, and football. When clicking any one of these icons, a screen will appear.

**Figure 10:**
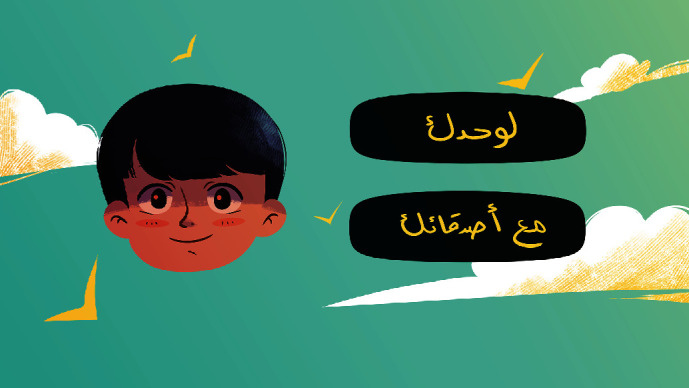
Sports' playing options.

Figure 10 is the screen for selecting the sports playing options. On this screen, the player can choose to play alone or with challenging friends that the player chooses.

**Figure 11:**
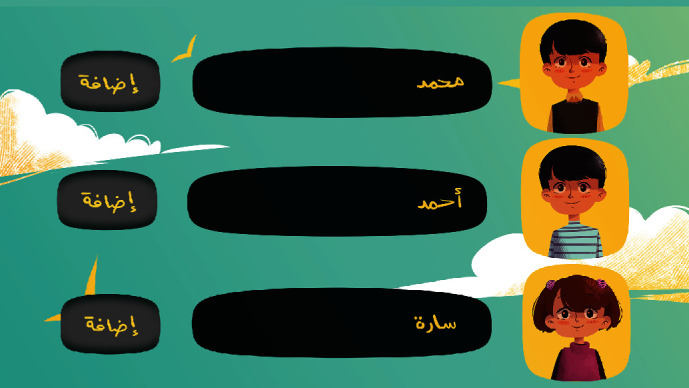
Friends' list screen.

Figure 11 is the friends' list screen that will appear if the player chooses to play with challenging friends. By clicking the Add button, the player can add the friends they want to challenge.

**Figure 12:**
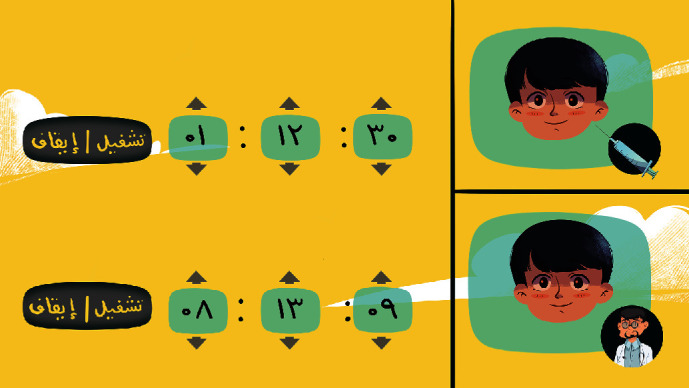
Reminder screen.

Figure 12 shows the screen that will appear when the player clicks the appointment icon on the home screen. This screen has two reminders. The player can set the time of the reminder by scrolling the clock and clicking the ON button. There are two reminders on this screen, one for the doctor's appointments and one for the insulin injection time. After setting a reminder, a notification will appear on the home screen of the child's device.

**Figure 13:**
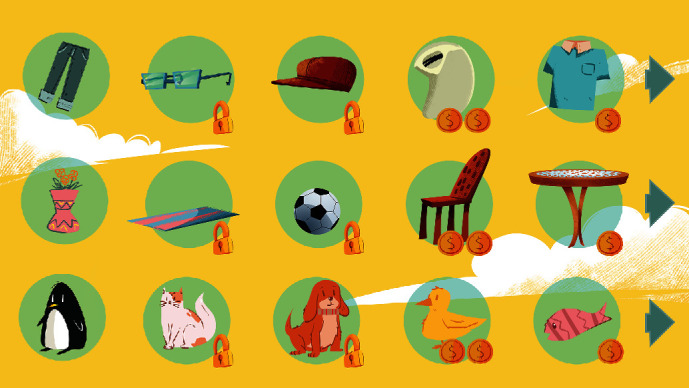
Shopping cart screen.

Lastly, Figure 13 illustrates the screen that will appear when the player clicks the shopping cart icon on the home screen. This screen shows the items that the player can buy using the accumulated points. The player can only buy the items that are available based on the level he\she reached. By moving to the next level, more items will be available to the player. The screen includes three types of items, clothes, pets, and accessories.

## 4. Discussion

The outcomes of this study indicated that it was possible to design the screens of a future gamified mobile application for the self-management of diabetes type 1 in children in Saudi Arabia based on the opinion of caregivers.

The main features of the designed screens were taking care of a character, the use of a friend challenge, the inclusion of rewarding principles, the use of reminders and notifications, and the inclusion of features to improve medication adherence, blood glucose readings, physical activities, and adoption of healthy eating habits.

Regarding taking care of a character, McCulloch et al. demonstrated that the use of a character makes the application more interesting and improves user-engagement with the application [26]. These authors suggested that the presence of a character in the application contributes to adherence to medication as well as to increase the blood glucose level readings [26]. Additionally, a recently published review showed that out of eight commercial-free applications, five of them used a character to be cared for by users (Commander Gage, Jerry the Bear, Pandabetic, Diapets, and Glucozor) [25]. As suggested by nearly a third of the caregivers, this feature was incorporated in the design of the screens of the gamified application proposed in this study.

Also, more than half of the caregivers (61.5%) indicated that the friend challenge was a favorable feature to be included in the design of the application. Therefore, this feature was included in the design of the screens. A study conducted by Stone suggested that the use of a friend challenge feature improves the self-management of diabetes condition through the competition concept [27]. It is pertinent to comment that no commercial application of the aforementioned literature review included a friend challenge function [25].

Based on the questionnaire survey distributed to the caregivers, the most favorable rewarding principle to include in the design of the screens was levels and leaderboard. The leaderboard was included as a rewarding principle as the friend challenge feature is best used with the leaderboard rewarding principle [28]. According to a study carried out by Cafazzo et al., the use of rewarding principles increases the frequency of blood glucose readings [29]. About the commercial applications reviewed in the study mentioned above, the most widely used rewarding principle was points [25]. This principle was used in three commercial applications: Jerry the Bear, Pandabetic, Diapets, and Glucozor [25].

Concerning the use of reminders and notifications, more than half of the caregivers agreed that this feature was useful for children. Consequently, this function was included in the design of the screens to make the application more effective in managing the diabetes condition. On this topic, Cafazzo et al. showed that the use of reminders increased the frequency of blood glucose readings [29].

Furthermore, the designed screens presented some features to improve the health monitoring behaviors of diabetic children including improving medication adherence, increasing the blood glucose readings, supporting physical activities, and helping to adopt healthy eating habits. Several studies have included these features in the design of applications for the self-management of type 1 diabetes in children [25, 26, 29, 30].

In general, the screens of the gamified app were designed to encourage children with diabetes type 1 diabetes to self-manage their health status by themselves.

About the suitability of the application screens for the age of the children involved in this study, it is pertinent to mention that the screens were designed based on the general characteristics of commercial applications developed for this age range [25].

The main limitation of this study was the impossibility of implementing the designed screens due to the end of the study period. Likewise, another limitation caused by the short time available to carry out the research was the small sample size of the participants in the survey (65 participants). Regarding this issue, the statistically significant sample size for the analysis to support the results had to be 80 respondents [31]. Similarly, the lack of a formal statistical validation method of the questionnaire, the lack of a statistical analysis of the results, and the absence of a theoretical approach to guide the research were important shortcomings of this study. Furthermore, the fact that the caregivers were not asked together with their children to develop the game strategies to design the screens and the possibility that the study was biased because the largest proportion of caregivers were female and participants with high educational level were other drawbacks of this study.

Future studies will be directed to design and implement a future gamified application for self-management of type 1 diabetes in children in Saudi Arabia. Furthermore, a clinical trial to test the effectiveness of the future application will be carried out.

## 5. Conclusion

In this study, the screens of a future gamified mobile application for self-management of type 1 diabetes in children in Saudi Arabia were designed based on the opinion of caregivers.

The main theme of the game designed on the screens was the care of a diabetic character (boy or girl). Also, various features such as challenging friends, sports activities, notifications, reminders, and rewards were incorporated into the game dynamics. The practical implementation of the future mobile application can motivate children with diabetes to improve eating habits, physical exercise, and cognitive, emotional, and social behaviors to maintain a stable state of health. It also can help to monitor blood glucose readings and adhere to medication treatment. The designed screens are adapted to the Arab culture.

## Figures and Tables

**Table 1:** Demographic information of caregivers (*n* = 65).

Variable	n (%)
Gender	
Female	64 (98.5%)
Male	1 (1.5%)
Age (years)	
20	2 (3.1%)
21-30	17 (26.2%)
31-40	28 (43.1%)
41-50	16 (24.6%)
51-60	2 (3.1%)
Level of education	
College degree	39 (60%)
High school	23 (35.4%)
Secondary school	3 (4.6%)
Primary	0 (0%)
Kinship relation with the diabetic child	
Mother	47 (72.3%)
Father	1 (1.5%)
Siblings	6 (9.2%)
Other	11 (16.7%)

**Table 2:** Demographic information of children with diabetes (*n* = 65).

Question	*n* (%)
What is the gender of your child?	
Male	30 (46.2%)
Female	35 (53.8%)
How old is your child? (years)	
7-8	13 (20%)
9-10	13 (20%)
11-12	20 (30.8)
Other	19 (28.9%)
When your child was diagnosed with diabetes? (years)	
<1	10 (15.4%)
1-3	24 (36.9%)
4-6	21 (32.3%)
Other	10 (15.2%)

**Table 3:** Opinion of caregivers of children with diabetes about the design of the screens of the mobile application (*n* = 65).

Question	*n* (%)
In your opinion, what is the most appropriate design of the game for your diabetic child?	
Questions and answers	16 (24.6%)
Runner game (ex: Crash game)	16 (24.6%)
Taking care of a character	19 (29.2%)
Stories	13 (20%)
Others (information arranged by alphabetical characters)	1 (1.5%)
In your opinion, what is the most appropriate rewarding style that shall be used in the game for your diabetic child?	
Points	32 (49.2%)
Levels	40 (61.5%)
Leaderboards (ranking of the players in the game)	18 (27.7%)
What are the features that you would like to have in the game for your diabetic child?	
Reminders and notifications	35 (53.8%)
Social interaction	25 (38.5%)
Friends challenges	40 (61.5%)
What are the most behaviors that you struggle to change of your child to manage his\her condition?	
Physical activities	17 (26.2%)
Managing the medications	10 (15.4%)
Nutrition problems	40 (61.5%)
Monitoring the blood glucose level	36 (55.4%)
Other (mental issues)	1 (1.5%)

**Table 4:** Level of utility of the application (*n* = 65).

Please rate on a scale from 0-10, how much would your diabetic child benefit from an application designed to manage his\her diabetes condition?	*n* (%)
0(not helpful)	1 (1.5)
1	1 (1.5)
2	0 (0.0)
3	0 (0.0)
4	2 (3.1)
5	13 (20.0)
6	5 (7.7)
7	4 (6.2)
8	9 (13.8)
9	5 (7.7)
10 (very helpful)	25 (38.5)

## Data Availability

Data available on request.

## Supplementary Materials

Supplementary Materials Appendix: guardian's perspective towards designing a mobile application for T1 diabetic children.
