# Supplementary material for: Caregiver's Opinions on the Design of the Screens of a Future Gamified Mobile Application for Self-Management of Type 1 Diabetes in Children in Saudi Arabia
Source: Int J Telemed Appl. 2021 Feb 4;2021:8822676. doi: 10.1155/2021/8822676 (PMC7880719; doi:10.1155/2021/8822676)
Supplement: Supplementary Materials — Appendix: guardian's perspective towards designing a mobile application for T1 diabetic children. [file 8822676.f1.pdf]

Guardian's Perspective Towards Designing a Mobile Application for T1 Diabetic Children

جامعة الإمام عبدالرحمن بن فيصل
IMAM ABDULRAHMAN BIN FAISAL UNIVERSITY

Demographic Information of Guardian

1* Age:

- [ ] Less than 20
- [ ] 21-30
- [ ] 31-40
- [ ] 41-50
- [ ] 51-60
- [ ] 61-70
- [ ] Other (Please Specify)

2* Gender:

- [ ] Male
- [ ] Female

3* Level of education:

- [ ] Primary
- [ ] Secondary
- [ ] High school
- [ ] College degree
- [ ] Other (Please Specify)

4* what is your kinship relation with the diabetic child:

- [ ] Mother
- [ ] Father
- [ ] Siblings
- [ ] Other (Please Specify)

Demographic Information of the Diabetic Child

5* When your child was diagnosed with diabetes:

- ☐ <1year
- ☐ 1-3 Year
- ☐ 4-6 Year
- ☐

Other (Please Specify):

6* How old is your diabetic child:

- ☐ 7-8 Year
- ☐ 9-10 Year
- ☐ 11-12 Year

7* What is the gender of your diabetic child:

- ☐ Male
- ☐ Female

Mobile Application Information

8* In your opinion, how much your diabetic child would benefit from an application that is designed to manage his\her condition:

0	1	2	3	4	5	6	7	8	9	10
Less helpful										More helpful

9* In your opinion, what's the most appropriate design of the game for your diabetic child:

- ☐ Questions & Answer
- ☐ Runner game
- ☐ Taking care of a character
- ☐ Stories
- ☐

Other (Please Specify)

10* **In your opinion, what's the most appropriate rewarding style that shall be used in the game for your diabetic child:**

- [ ] Points
- [ ] Levels
- [ ] Leaderboards
- [ ] Other (Please Specify)

11 What are the features that you would like to have in the game for your diabetic child:

- [ ] Reminders and notifications
- [ ] Social interaction
- [ ] Friends challenges
- [ ] Other (Please Specify)

12 What are the most behaviors that you struggle to change of your child to manage his\her condition:

- [ ] Physical activities
- [ ] Managing the medications
- [ ] Nutrition problems
- [ ] Monitoring the blood glucose level
- [ ] Other (Please Specify):

13 Any additional comment:

Thank You For Completing This Survey
